# Recent advances of engineered probiotics for therapeutic applications

**DOI:** 10.1016/j.bidere.2025.100039

**Published:** 2025-07-15

**Authors:** Lu Zhang, Na Chen, Haofeng Chen, Chaoqun Tang, Junyi Wang, Yan Wang, Yang Zhang, Hao Guo, Jifeng Yuan

**Affiliations:** School of Life Sciences, Faculty of Medicine and Life Sciences, Xiamen University, Fujian, 361102, China

**Keywords:** Microbiota, Disease treatment, Engineered probiotics, Synthetic biology

## Abstract

A great number of multifactorial diseases, including neoplastic, metabolic, and autoimmune diseases, have been associated with microbiota dysbiosis. Recently, there has been an increasing understanding of the importance of microbiome and their impact on human health. Advances in synthetic biology have led to the development of probiotics as diagnostic tools and disease treatment approaches. In this review, we briefly summarize recent examples of engineered probiotic-based therapeutics in human diseases, including cancers, gastrointestinal disorders, infectious diseases, and metabolic disorders. Finally, we discuss the challenges and opportunities in developing engineered probiotics for disease treatments.

## 1 Introduction

The World Health Organization (WHO) defines health as complete physical, mental, and social well-being, not just the absence of disease or infirmity [1]. The microorganisms in the oral cavity and saliva are screened by the digestive system and settle in the intestine, forming a complicated and energetic intestinal microbiota ecosystem, in which there are a wide variety of microorganisms, including bacteria, fungi, archaea and viruses, showing significant spatial heterogeneity [2,3]. The microbiota is actively involved in diverse physiological processes within the human body, and participates in the regulation of physiological functions such as immunity, metabolism, pathogen defense and mucosal protection by releasing active substances including short chain fatty acids (SCFAs), lipopolysaccharide (LPS) and bile acids (BAs), and acts as a key bridge between the gut and the brain by the microbial-brain-gut axis. Beyond their natural presence in the human body, the naturally-isolated probiotics have been commonly used in the pharmaceutical and food industry. For instance, probiotics are added into various foods [4] such as yogurt (including lactic acid bacteria and *Bifidobacterium*), Kefir, Kimchi (including lactic acid bacteria) and natto (including *Bacillus subtilis*). Among these probiotics - enhanced foods, yogurts fortified with *Bifidobacterium* and other beneficial strains are especially popular among consumers, as they are a convenient and delicious way to introduce beneficial microorganisms into the diet. *Lactobacillus* and *Bifidobacterium* have been used to regulate the bowel functions and probiotic medications are prescribed as adjunct therapy for antibiotic-associated diarrhea to restore gut microbiota balance. The link between intestinal microbiota structural changes and disease pathogenesis is a complex research field. Gut microbiota dysbiosis refers to microbiome structural and/or functional changes, often shown by lower α-diversity (species richness), reduced beneficial microbes (e.g., *Firmicutes*, *Bacteroidetes*), or altered taxonomic composition (β-diversity).It is commonly related to an elevation in the relative abundance of the phylum *Proteobacteria* (e.g., the family *Enterobacteriaceae*), often exceeding 10 ​% of the total microbiome [5,6]. Taking *Faecalibacterium prausnitzii* as an example, its population is extremely low in early infancy and rises following the establishment of primary colonizing bacteria. However, decreased counts of *F. prausnitzii* have been documented in multiple inflammatory bowel diseases (IBDs), colorectal cancer and type 2 diabetes [7]. And *Akkermansia muciniphila*, considered a promising next-generation probiotic, has been observed to exhibit reduced levels in patients with IBD [8].

**Fig. 1:**
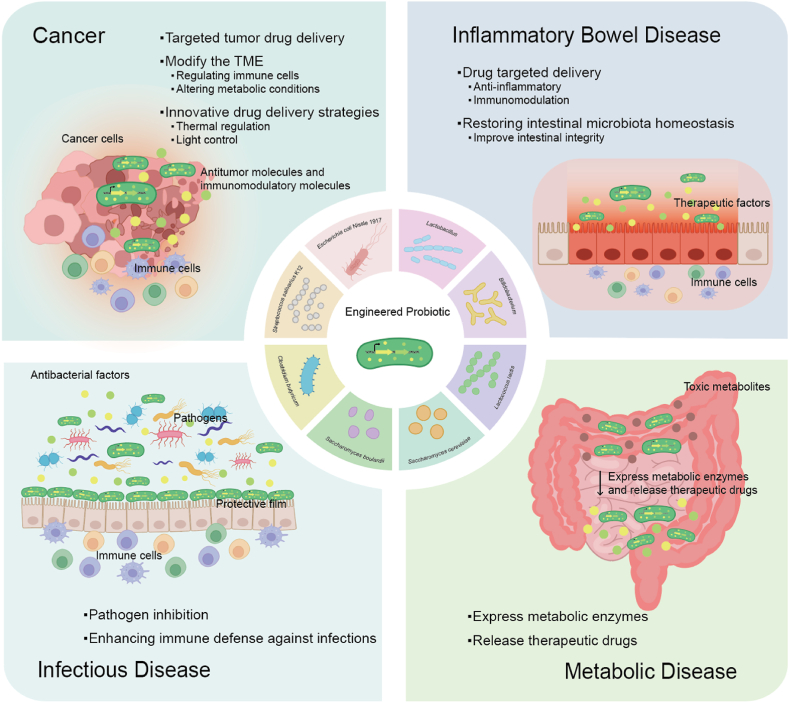
**Summary of functional mechanisms of engineered probiotics.** The mechanisms by which engineered probiotics exert therapeutic effects can be summarized into four aspects: (1) as delivery vehicles for therapeutic molecules: Secreting therapeutic or immunomodulatory factors (such as cytokines, enzymes, and antimicrobial peptides) for targeted treatment; (2) as assistants for host metabolism: Providing metabolic assistance to alleviate disorders like phenylketonuria (PKU) and hyperammonemia; (3) as defenders of gut microenvironmental homeostasis: colonizing and modulating the gut microenvironment by competing for nutrients, secreting SCFAs to regulate the immune system, and maintaining the intestinal barrier; (4) as tracking missiles for tumor elimination: enabling targeted oncology therapy by delivering anti-cancer agents and activating the immune system.

With the advancement of synthetic biology, engineered probiotics are emerging for effectively treating many human-related diseases (Fig. 1). Although engineered probiotics have enormous potential in medical applications, researchers need to consider long-term efficacy of engineered probiotics and the effect of their releases to environment. For instance, when selecting selectable markers, both screening efficiency and safety must be taken into account. Considering the issue of horizontal transfer of antibiotic resistance genes, antibiotic screening markers are not suitable for screening engineered microorganisms used in medical purposes [9]. Therefore, non-resistant markers with higher safety, such as auxotrophic markers, are more preferable. Meanwhile, the inducers for gene expression in engineered probiotics should effectively initiate the expression of target genes without causing harm to the human body. In this review, we briefly summarize the recent cases related to the treatment of human diseases, including cancers, gastrointestinal disorders, infectious diseases, and metabolic disorders. By analyzing existing research results, this review provides references for future research directions and explores methods to overcome current limitations and challenges to promote the widespread application of engineered probiotics in medical treatment.

### 1.1 Engineered microbes for cancer treatment

The occurring of cancer has been escalating in the past decades due to the population aging, socioeconomic development, and life style changes [10]. As early as 2010, Forbes et al. summarized the use of genetically edited bacteria for treating cancer [11]. Afterwards, an increasing number of researchers noticed the research direction of engineered probiotics, and studies and reviews on engineered probiotics have continued to emerge. These advancements not only deepen the understanding of probiotic mechanisms but also broaden their potential in clinical therapy. Summarizing the content of these studies, they can be broadly categorized into three groups. The first category leverages the ability of engineered probiotics to colonize tumors, aiming to deliver therapeutic drug molecules directly to the tumor site. The second category focuses on modifying the tumor microenvironment (TME) using engineered probiotics, such as regulating immune cells or altering metabolic conditions. The third category of research centers on optimizing and innovating the delivery methods of engineered probiotics.

Probiotics are engineered to secrete active substances (such as immunomodulatory proteins, prodrug-converting enzymes, cytokines, nanobodies, small interfering RNAs, α-hemolysin, and toxins) in TME [12]. These engineered probiotics and their payloads work together to reshape the TME [12]. Back in 2004, S. L. Young et al. employed recombinant *Mycobacterium smegmatis* for tumor therapy; using this bacterium as a vector for cytokine delivery offers an advantage due to its provision of additional adjuvant activity [13]. In Takayuki Sasaki’ study, recombinant *Bifidobacterium longum* expressing cytosine deaminase (eCD) is injected into tumor cells of breast cancer mice, and eCD can convert the administered 5-fluosrocytosine (5-FC) to 5-fluorouracil (5-FU), significantly increasing intratumoral 5-FU level to reduce tumor size [14]. By tail vein injection of engineered *B. longum* in breast cancer mice, *B. longum* could selectively colonize the tumor hypoxic microenvironment [14]. Candice and coworkers engineered *Escherichia coli* Nissle 1917 (EcN) to release cytokines, and oral administration of this engineered EcN was demonstrated to target adenomas and function in mouse models. At the neoplastic site, the engineered EcN delivered blocking nanobodies, and oral administration of this strain reduced adenoma burden by ∼50 ​% [15]. Chun Loong Ho et al. reprogrammed the EcN to bind to the heparan sulfate proteoglycan (HSPG) on the cancer cell surface and to secrete myrosinase for the conversion of glucosinolate to sulforaphane [16], which promotes apoptosis in cancer cells and results in colorectal tumor clearance in murine models [16]. However, due to the low level of dietary glucosinolate from vegetables, it is not a practical approach for colorectal tumor treatment. Sheng-Nan Jiang and colleagues genetically engineered *E. coli* strain K12 to synthesize the cytotoxic protein cytolysin A (ClyA) for tumor cell killing, while the bacterial luciferase (Lux) operon was employed for imaging infected tissues. They found that this genetically engineered strain can inhibit tumors and a combination of targeted therapy and radiotherapy substantially reduces the rate of tumor growth in mice [17].

Another approach is to utilize engineered probiotics to modify the TME, including immune status and metabolic conditions. Since stimulator of interferon genes may impair the establishment of antitumor immunity, Daniel S. Leventhal designed engineered EcN to target STING pathway activation to antigen-presenting cells (APCs) in TME. The engineered EcN triggers complementary proinflammatory pathways through pattern-recognition receptors (PRRs) activation [18]. In another example, Thomas M. Savage et al. engineered EcN to intratumorally secrete chemokines, which attract adaptive immune cells into the TME, recruit innate immune cells [19]. The metabolic conditions in the TME play a crucial role in tumor progression, so inhibiting tumors can be achieved by altering these metabolic conditions. Fernando et al. deleted the arginine repressor gene (*ArgR*) and integrated the N-acetylglutamate synthase gene (*ArgA*), the first enzyme in the arginine biosynthesis pathway, into the non-pathogenic strain EcN. The engineered EcN continuously converts ammonia accumulated in tumors into L-arginine, increases the number of tumor-infiltrating T cells, and exhibits marked synergistic effects with programmed cell death ligand 1 (PD-L1) blocking antibodies in tumor clearance [20]. Cancer cells primarily generate ATP through the glycolytic process. By competing for glucose in the TME, glycolysis in tumor cells can be blocked, thereby killing tumor cells. Penghao Ji et al. engineered *E. coli* MG1655 to express exogenous glucose dehydrogenase (GDH) to competitively deplete glucose in tumor regions and confirmed that it consumes glucose and trigger apoptosis of tumor cells [21].

With the continuous deepening of researchers' understanding of engineered probiotics and tumor therapy, along with the ongoing advancement of engineered probiotic editing technologies, scientists are no longer confined to simply using these microorganisms for therapeutic molecule delivery. Instead, they are actively improving and innovating drug delivery strategies with engineered probiotics. Lianyue Li et al. engineered EcN to respond to thermal stimuli and produce tumor necrosis factor α (TNF-α), thereby inhibiting tumor growth [22]. The team led by Mohamad H. Abedi developed engineered probiotics that can continuously generate and release immune checkpoint inhibitors during ultrasound hyperthermia [23]. Scholars including Xiaoqiang Zhu modified EcN such that under 808 ​nm light irradiation, lanthanide upconversion nanoparticles (UCNPs) emit blue light to activate EcN for secreting flagellin B (flaB) [24]. The team of Meiyang Yang developed engineered probiotics with surfaces anchored by polyethylene glycol-polyethyleneimine-citraconic anhydride nanoparticles (PNPs), which are loaded with human sulfatase 1 (hsulf-1) enzyme plasmid and doxorubicin (DOX). These engineered probiotics can actively target and colonize tumor sites, achieving explosive concentrated release of silver (Ag) through lytic genes [25]. Additionally, Longliang Qiao et al. developed engineered probiotics that can be induced by near-infrared (NIR) light to express PD-L1/cytotoxic T lymphocyte-associated protein 4 (CTLA-4) nanobodies or azurin and cytolysin A at tumor sites [26]. These innovations highlight the evolving landscape of engineered probiotic-based drug delivery, where precise spatiotemporal control and multifunctional design are driving progress in tumor therapy.

Overall, a number of representative means of using engineered probiotics to treat cancer can be reflected in Table 1.

**Table 1:** Research on engineered probiotics for cancer therapy in 2021–2025.

Chassis cells	Engineering strategy	Ref.	Year
*Pediococcus pentosaceus*	Engineered with dual gene cassettes: one encoding the therapeutic protein P8 fused to a secretion signal peptide, and the other a complementation system.	[27]	2021
*Enterococcus faecalis*	Expresses and secretes the NlpC/p60 peptidoglycan hydrolase SagA to generate immunologically active muropeptides.	[28]	2021
EcN	Lacks the *ArgR*, enabling it to continuously convert the ammonia accumulated in tumors into L-arginine.	[20]	2021
EcN	Responds to thermal stimuli and produces TNF-α to inhibit tumor growth.	[22]	2022
EcN	Continuously produces and releases immune checkpoint inhibitors after focused ultrasound hyperthermia is applied.	[23]	2022
EcN	Loaded with magnetic nanoparticles to achieve magnetic field guidance and magnetothermal ablation, combined with thermally regulated expression of fluorescent proteins and type II NADH: quinone oxidoreductase (NDH-2).	[29]	2022
*E. coli*	Has a negatively charged surface that can specifically target hypoxic tumor regions and couple with cationic lipid nanoparticles (PTX-CLs) loaded with paclitaxel (PTX) and perfluorohexane (PFH) via electrostatic adsorption.	[30]	2022
*E. coli* MG1655	Expresses the immunomodulatory molecule interferon-γ (IFN-γ) under brief hyperthermia induced by focused ultrasound.	[31]	2022
EcN	Blue light induces lysis at the tumor site and releases tumor apoptosis-inducing ligand (TRAIL).	[32]	2022
*L. lactis*	Expresses a fusion protein of Fms-like tyrosine kinase 3 ligand and co-stimulatory molecule OX40 ligand at the tumor site, converting "cold" tumors into "hot" tumors.	[33]	2022
*Lactobacillus rhamnosus* GG	Under ultrasound irradiation, the CRISPR system generates reactive oxygen species (ROS) to induce immunogenic cell death (ICD), while ROS disrupts endosomes/lysosomes to release Cas9/sgRNA for knocking out the *IDO1* gene in tumor cells, thus reversing tumor immunosuppression.	[34]	2022
*E. coli* MG1655	Expresses exogenous GDH to competitively deplete glucose in tumor regions.	[21]	2023
Lactic acid bacteria	Produces anti-PD-L1 single-chain variable fragments (scFv) conjugated with green fluorescent protein (GFP).	[35]	2023
EcN	Under 808 ​nm photoirradiation, lanthanide UCNPs emit blue light to activate EcN for the secretion of flaB.	[24]	2023
EcN	Expresses the activating mutant of human chemokine CXCL16 (hCXCL16K42A) or CCL20 to attract adaptive immune cells into the TME.	[19]	2023
*E. coli*	Loaded with Fe_3_O_4_@lipid nanocomposites, it allows constant magnetic field-controlled motion for tumor targeting, converts alternating magnetic field signals into heat to trigger lysis protein expression, and releases anti-CD47 nanobodies.	[36]	2023
*Staphylococcus epidermidis*	Expresses tumor antigens anchored to secreted or cell-surface proteins.	[37]	2023
EcN	Utilizes its siderophore-mediated metal uptake pathway to selectively enrich copper radionuclides chelated with yersiniabactin (YbT), with^64^Cu for positron emission tomography (PET) imaging and^67^Cu for delivering cytotoxic doses.	[38]	2023
EcN	Produces cyclic dinucleotides to activate the STING pathway under hypoxic conditions.	[39]	2023
EcN	Secretes biologically active IL-2 (mi-IL2).	[40]	2023
EcN	Releases synthetic targets that label tumor tissues in situ to guide chimeric antigen receptor (CAR)-T cell recognition and killing, while co-secreting chemokines.	[41]	2023
EcN	Loaded with tumor antigens and trained immunity inducer β-glucan to form a personalized cancer vaccine (such as BG/OVA@EcN).	[42]	2024
EcN	Highly expresses tyrosinase to generate Cy5-labeled melanin-like polymers, possessing both fluorescent imaging and NIR photothermal effects.	[43]	2024
EcN	Colonizes colorectal tumors, produces salicylate as a detection marker, and locally releases therapeutic molecules such as GM-CSF, anti-PD-L1, and CTLA-4 nanobodies.	[15]	2024
EcN	Integrates genetic circuits such as XOR gate-amplified genetic switches to detect TME markers and modulate therapeutic payload expression.	[44]	2024
EcN	Secretes anti-PD-1 antibodies and IL-2.	[45]	2024
*E. coli*	The surface is anchored with PNPs loaded with hsulf-1 enzyme plasmid and DOX. It colonizes tumor sites, and achieve explosive concentrated release of Ag through lytic genes.	[25]	2024
*E. coli* MG1655	Carries an acoustic reporter gene and the hyperthermia-responsive IFN-γ gene, and is chemically modified with DOX.	[46]	2024
*L. plantarum*	Surface-loaded with anticancer prodrugs, which are activated by tumor signals to release 7-ethyl-10-hydroxycamptothecin (SN-38) at the tumor site.	[47]	2024
*Salmonella*	Accumulates at the tumor site, continuously secrete nattokinase to inactivate cancer-associated fibroblasts.	[48]	2024
EcN	Expresses anti-PD-L1 and anti-CD9 nanobodies, is modified with zinc-based metal-organic frameworks (ZIF-8CHO) loaded with indocyanine green (ICG), and can undergo spatiotemporally specific lysis to release nanobodies in response to NIR radiation.	[49]	2024
*Salmonella typhimurium* VNP20009	Conjugated with calcium carbonate nanoparticles to target and deliver curcumin to tumor sites, where curcumin synergistically enhances intracellular calcium overload to induce mitochondrial dysfunction and apoptosis in tumor cells.	[50]	2024
EcN	Delivers neoepitope-containing peptide arrays to tumor sites and enhances susceptibility to blood clearance and phagocytosis.	[51]	2024
*E. coli*	One strain synthesizes the photosensitizer precursor 5-aminolevulinic acid (5-ALA), and another strain is endowed with the ability to photoregulate the release of PD-L1 nanobodies.	[52]	2024
*C. butyricum*	Conjugated via matrix metalloproteinase-2 (MMP-2)-responsive peptides to deliver vactosertib (VAC) and gemcitabine (GEM) to the tumor core while competitively reducing the abundance of γ-proteobacteria.	[53]	2025
*S. boulardii*	Delivers immune checkpoint inhibitors to intestinal tumors.	[54]	2025
*Magnetospirillum gryphiswaldense*	Surface-conjugated drug-loaded nanoliposomes for targeted tumor drug delivery.	[55]	2025
EcN	Expresses adenosine deaminase on its surface under hypoxic conditions, targets tumors, converts immunosuppressive adenosine to inosine.	[56]	2025
*Lactobacillus acidophilus*	Coated with a polymeric stealth coating and co-loaded with lactate oxidase (LOx) and horseradish peroxidase (HRP) to achieve deep tumor drug delivery.	[57]	2025
EcN	Delivers interleukin-32 (IL32) and selenium nanoparticles to tumor sites.	[58]	2025
EcN	Expresses the nanobody Nb215 and is conjugated with the photothermal dye indocyanine green (ICG) to enable photothermal therapy (PTT).	[59]	2025
*Salmonella enteritidis*	NIR light can induce it to express PD-L1 and CTLA-4 nanobodies or azurin and cytolysin A at tumor sites.	[26]	2025
EcN	Colonizes tumors and locally releases interferon-γ.	[60]	2025
EcN	Continuously converts ammonia into L-arginine to increase intratumoral L-arginine concentration, while the polydopamine (PDA) on its surface generates heat under NIR light irradiation to ablate tumor cells.	[61]	2025

### 1.2 Engineered microbes for alleviating inflammatory bowel disease (IBD)

IBD is a chronic non-specific inflammatory bowel conditions manifesting as abdominal pain diarrhea and blood in the stool, which severely impacts the daily life of patients. [62]. Similar to secreting active substances to treat cancer, the modified probiotics overexpress and release the therapeutic factor such as immune cytokines, antioxidant peptides, antimicrobial peptides SCFAs or organic acids (Table 2), which function to regulate pro-inflammatory and anti-inflammatory cytokines, improving the integrity of the intestinal barrier, and regulating the intestinal microbiota.

**Table 2:** Representative engineered probiotics for the IBD therapy in 2021–2025.

Chassis cells	Engineering strategy	Ref.	Year
*S. cerevisiae*	Expresses a human P2Y purinoceptor 2 (P2Y2) to sense the pro-inflammatory molecule eATP and secretes the ATP-degrading enzyme apyrase to achieve a self-regulated neutralizing response.	[65]	2021
EcN	Continuously synthesizes 3HB in the intestines of mice with colitis, increasing the levels of intestinal 3HB and short-chain fatty acids.	[63]	2021
EcN	Expresses catalase and superoxide dismutase, and is coated with chitosan and sodium alginate to improve its bioavailability in the gastrointestinal tract.	[66]	2022
EcN	Senses the level of the inflammatory biomarker thiosulfate to activate a base-editing system, generating heritable genomic DNA sequences and producing colorimetric signals for noninvasive, real-time monitoring and recording of the occurrence and progression of IBD. Concurrently, it drives the tunable release of the immunomodulator AvCystatin.	[67]	2023
*E. coli*	Secretes functional single-domain antibodies, nanobodies, stably colonizes the mouse intestine and secretes TNF-α-neutralizing nanobodies.	[68]	2023
EcN	The two strains respectively express and secrete anti-TNF-α nanobody and IL-10.	[69]	2024
EcN	Expresses secretable IL-2, an immunomodulator, and is coated with the enteric coating material Eudragit L100-55.	[70]	2024

SCFAs play a pivotal role in maintaining intestinal homeostasis and modulating inflammatory responses in IBD. Xu Yan et al. successfully constructed (R)-3-hydroxybutyrate (3HB) producing EcN strain, which can alter the SCFA concentration and enrich the probiotic abilities, reinforcing the amelioration of colitis. The EcN and 3HB could be combined to exert a synergistic effect on improving the IBD treatment [63]. Liu et al. constructed recombinant *Saccharomyces boulardii* that secretes different proteins including the anti-inflammatory protein interleukin-10 (IL-10), tumor necrosis factor receptor 1 extracellular domain, atrial natriuretic peptide (ANP) and alkaline phosphatase. ANP secreting strain can effectively alleviate dextran sulfate sodium (DSS) induced colitis in mice, which are manifested as downregulation of TNF-α, interleukin-1β (IL-1β) and upregulation of interleukin-6 (IL-6) in colon tissues [64]. Combined directed evolution and synthetic biology approaches, an engineered *Saccharomyces cerevisiae* to sense extracellular adenosine triphosphate (eATP) and secrete the cluster of differentiation 39 (CD39) like eATP-degrading enzyme apyrase was reported to alleviate IBD in the animal model [65]. These studies highlight the diverse strategies leveraging microbial engineering to target SCFA metabolism, protein secretion, and environmental sensing, paving the way for innovative therapeutic approaches in IBD management.

### 1.3 Engineered probiotics for curing metabolic diseases

Engineered probiotics provide an important direction for the metabolic disease's treatment. Gut microbiota can regulate various host-related metabolic reactions and generate metabolites including SCFAs, bile acids, and choline which are vital for host health; the imbalance of intestinal microbes and their metabolites can lead to obesity and related metabolic diseases [71]. The application of engineered probiotics is investigated for several kinds of metabolic disease including diabetes, obesity, hyperammonemia, and phenylketonuria (Table 3).

**Table 3:** Representative engineered probiotics for the treatment of metabolic diseases in 2021–2025.

Disease category	Chassis cells	Engineering strategy	Ref.	Year
Diabetes	*L. lactis*	Carrying plasmids encoding IL-4 and IL-10.	[80]	2021
Diabetes	*L. plantarum*	Persistently expresses GLP-1.	[78]	2023
Obesity	*S. boulardii*	Secretes Exendin-4 in the gastrointestinal tract.	[85]	2023
Hyperammonemia	EcN	Converts ammonia to arginine and synthesizes butyrate.	[90]	2021
Phenylalanine	EcN	Expresses Phe-metabolizing enzymes in response to anoxic conditions.	[94]	2021
Phenylalanine	EcN	Expresses the Phe-metabolizing enzyme PAL.	[95]	2021
Phenylalanine	EcN	Expresses PAL as an intracellularly free and a cell surface-immobilized enzyme.	[96]	2022

#### 1.3.1 Diabetes

Diabetes is a chronic disease, including type I diabetes (T1DM) and type II diabetes(T2DM). Due to insulin deficiency, the glucose levels in the human blood are higher than the normal range [72]. In diabetic patients, the intestinal structure had undergone significant changes, such as the imbalance of the ratio of *Firmicutes* and *Bacteroidetes*, the relative increase of *Firmicutes* and the relative decrease of *Bacteroidetes*, which are closely related to the pathogenesis of diabetes [73]. Based on this knowledge, scientists are using engineered probiotics to treat diabetes.

Glucagon-like peptide-1 (GLP-1), a second incretin peptide, potently stimulates glucose-dependent insulin secretion. Besides its insulinotropic effects, GLP-1 inhibits gastric emptying and glucagon secretion, decreases food intake, and slows the rate of glucose production [74], all of which should help to lower blood glucose in T2DM [75]. Thus, GLP-1 could be used as a treatment medicine for T2DM. However, traditional oral administration of GLP-1 will be damaged by the gastrointestinal environment and lose its effect. It was recently discovered that *A*. *muciniphila* secrete a GLP-1-inducing protein that improves glucose homeostasis and ameliorates metabolic disease in mice [76]. Scientists applied recombinant *L*. *lactis* [77] and *L*. *plantarum* [78] to deliver GLP-1 to the gut in mouse models. Meanwhile, the mutant GLP-1 with a higher half-life has been developed and expressed in the gut using *L*. *lactis* and verified for therapeutic efficacy.

The main cause of T1DM is that the body's own immune system attacks pancreatic β cells, resulting in insufficient insulin secretion. Some studies have engineered probiotics to express the islet β-cell antigen mainly recognized by the immune system or cytokine, stimulating the intestinal mucosal immune response, thus inducing the body's tolerance to pancreatic β cells. For example, researchers genetically modified *L. lactis* to deliver glutamic acid decarboxylase 65 (amino acids 370–575) and IL-10 [79] or interleukin-4 (IL-4) and IL-10 [80].

#### 1.3.2 Obesity

Obesity is defined as abnormal or excessive fat accumulation, and it is considered to be one of the most critical public health concerns in recent years [81]. Intravenous administration of synthetic oxyntomodulin (OXM) reduces food intake and body weight. R T Long et al. engineered *B. longum* to express OXM, and found that its inhibitory effects on food intake and body weight in overweight mice [82]. Low-carboxylated osteocalcin (ucOC) acts as a hormone to regulate carbon and energy metabolism. Fu Namai et al. engineered *L. lactis* to express mouse ucOC, and found it can trigger GLP-1 secretion to treat obesity [83]. W-Y Cao and his coworkers engineered *L*. *lactis* to overexpress bioactive human fibroblast growth factor 21 (FGF21) (a metabolism regulator), and found it reduced body weight of diabetes (Db/Db) mice through the activity of brown adipose tissue [84]. In another study, Hedin et al. engineered *S. boulardii* to secrete GLP-1 and Exendin-4. This *S. boulardii* successfully stimulated insulin secretion of wild-type mice. *In* *vivo* experiments have shown that it can significantly reduce the body weight of mice raised in a cold environment (8°C) and reduce food intake, thereby achieving the effect of treating obesity [85].

#### 1.3.3 Hyperammonemia

Hyperammonemia is a disease caused by the abnormal accumulation of ammonia in the human body. The metabolism of ammonia, and its subsequent excretion in the kidney, depends mainly on the normally functioning liver, and the ammonia from the portal circulation into the liver is mainly metabolized by the urea cycle enzymes [86]. Ammonia is mainly produced in the small intestine and colon and enters the systemic circulation [87]. Engineered probiotics that can colonize the gut can be used to deplete ammonia in the gut, thereby reducing ammonia accumulation and treating hyperammonemia. Back in 2008, Charles Nicaise et al. developed a *L*. *plantarum* strain overproducing alanine dehydrogenase, which can efficiently convert ammonia to alanine in three rodent models of hyperammonemia [88]. Caroline B Kurtz et al. engineered EcN to create the strain *SYNB1020*, which consumes ammonia in its environment and converts it to arginine. They found this strain improves hyperammonemia and survival in mice and shows dose-dependent exposure in healthy humans [89]. Rafael Ochoa-Sanchez et al. further modified *SYNB1020* to synthesize butyrate, a SCFA with anti-inflammatory/antioxidant properties that could protect the gut-barrier and brain function. This strain evaluated in bile-duct ligated (BDL) rats, and successfully attenuated hyperammonemia [90].

#### 1.3.4 Phenylketonuria

Phenylketonuria (PKU) stems from phenylalanine hydroxylase (PAH) deficiency: this enzyme converts phenylalanine (Phe) to tyrosine, a precursor for key hormones and skin/hair/eye pigments. PAH deficiency results in accumulation of Phe in the blood and brain, which further leads to neurological deficits and emotional and cognitive problems [91]. The main treatment of PKU is dietary restriction of Phe. However, long-term protein restriction may lead to malnutrition and a series of symptoms such as osteoporosis, anorexia, hair loss, and lethargy. Phe ammonia-lyase (PAL) exists in many plants, yeasts, fungi and *Streptomyces*, and can catalyze the deamination of L-Phe to produce trans-cinnamic acid. Therefore, this enzyme has a potential role in the diagnosis and treatment of PKU.

Ma xin et al. constructed an engineered probiotic strain EcN-PAL expressing Phe lyase through the Red homologous recombination system. The EcN-PAL strain could effectively degrade Phe in the culture medium under aerobic conditions and oxygen-deficient conditions. Long-term feeding of PKU mice with the engineered probiotic EcN-PAL reduces blood Phe levels to normal, suggesting that EcN-PAL degrades Phe in the intestinal tract or enhances its metabolic clearance, thereby controlling blood Phe concentration [92]. Vincent M Isabella et al. constructed *SYNB1618*, a Phe-degrading derivative of EcN*. SYNB1618* was engineered with two chromosomally integrated copies of *pheP* (a high-affinity Phe transporter) and three copies of *stlA* (the gene of PAL). Administration of *SYNB1618* to PAH enu mutation 2 mouse model of PKU reduced blood Phe concentration by 38 ​% compared with the control, independent of dietary protein intake [93]. The team later conducted the first human phase 1/2a study of the strain and demonstrated that *SYNB1618* was safe and well tolerated [94]. Subsequently, this team used a biosensor to screen out a more active PAL from the mutant PAL libraries, and engineered EcN to express this PAL. They found this strain outperforms *SYNB1618 in vitro* and *in vivo* [95]. Yu Jiang et al. achieved dual intracellular/extracellular Phe degradation in EcN by expressing free and cell-surface PAL, overcoming transport limitation. In *Pah* F263S PKU mice, this strain reduced blood Phe by 44.4 ​% vs. control EcN, independent of diet [96].

### 1.4 Engineered probiotics against infectious diseases

Bacterial infections represent the primary cause of morbidity globally. Indigenous probiotics suppress pathogens via mechanisms like antibacterial compound secretion, immune response modulation, nutrient/adhesion site competition, and inhibiting the expression of toxic proteins in intestinal pathogens [[97], [98], [99]]. A number of probiotic-based treatments have been developed to battle against infectious diseases caused by *Pseudomonas aeruginosa*, *Clostridioides difficile*, *Staphylococcus aureus*, etc. (Table 4).

**Table 4:** Representative engineered probiotics for the treatment of infectious diseases in 2020–2025.

Infection category	Chassis cells	Engineering strategy	Ref.	Year
*P. aeruginosa*	Lactic acid bacteria	Secretes enzymes to degrade *P. aeruginosa* biofilms.	[108]	2020
*C. difficile*	*S. boulardii*	Secretes antibodies that neutralize both TcdA and TcdB toxins.	[114]	2020
*C. difficile*	EcN	Restores bile salt metabolism in the gut during antibiotic-related dysbiosis.	[116]	2022
*C. difficile*	*L. lactis*	Secretes heterodimeric β-lactamase.	[118]	2022
*S. aureus*	*L. plantarum*	Senses *S. aureus* and releases lysostaphin protein.	[123]	2023
*L. monocytogenes*	*L. casei*	Expresses LAP from a non-pathogenic *Listeria* and a pathogenic *Listeria*.	[126]	2020
*S. pyogenes*	*S. salivarius* K12	Effectively releases antimicrobial agents by blocking GAS signal pathway.	[129]	2024

***P. aeruginosa*** is a multidrug-resistant pathogen that causes acute or chronic infections in immunocompromised individuals with conditions such as chronic obstructive pulmonary disease (COPD), cystic fibrosis, cancer, trauma, and burns [100]. *P. aeruginosa* can colonize the patient's respiratory and gastrointestinal tract, and is one of the leading causes of nosocomial infection [101]. Eradication of *P. aeruginosa* is challenging since *P. aeruginosa* has a remarkable capacity to resist antibiotics, which is explained by its extensive intrinsic resistome, low membrane permeability, and ability to form biofilms [102,103]. Several studies have reported engineered probiotics-based antimicrobial strategies which are highly effective in killing antibiotic resistant *P. aeruginosa*. A study published in 2011 developed an *E. coli* that can release pyocin S5 in the presence of *P. aeruginosa*, and confirmed that it can kill *P. aeruginosa in vitro* [104]. The researchers utilized the quorum sensing mechanism of *P. aeruginosa*, producing acyl homoserine lactones (AHLs) that induce engineered *E. coli* to express pyocin S5 and E7 lysis protein. The E7 lysis protein induces self-lysis of *E. coli* [105], thereby releasing pyocin S5. Pyocin S5 exhibits strong bactericidal activity against *P. aeruginosa* through membrane damage [106]. In 2017, this group modified this system in EcN by introducing an anti-biofilm enzyme gene, despersin B (DspB), which promotes the destabilization of mature biofilms to improve the microbial therapeutic efficacy and tested this modified system in *Caenorhabditis elegans* and murine infection models. The engineered EcN had prophylactic and therapeutic activity against *P. aeruginosa* during intestinal infection in two animal models [107]. In a followed study, the *Lactobacilli* were engineered to secrete enzymes to disrupt mature *P. aeruginosa* PA14 biofilms, further improving the efficacy of treating infection, which can degrade up to 85 ​% of *P. aeruginosa* biofilms [108].

***C. difficile*** infection (CDI) is a prevalent nosocomial infection that causes a range of diseases from mild diarrhea to fulminant colitis and death [109]. The widespread use of antibiotics leads to the emergence of high virulence and antibiotic-resistant strains, which increases incidence and treatment difficulty of CDI. Meanwhile, CDI is highly recurrent because *C. difficile* produces endospores that can remain dormant in the human gastrointestinal tract and are resistant to antibiotics [110]. Tissue damage and inflammation in CDI are mainly caused by two exotoxins, *C. difficile* toxin A (TcdA) and *C. difficile* toxin B (TcdB). Antibodies against both toxins can prevent and treat CDI in animal models [111,112], and currently allows parenteral infusion of purified antibodies to treat CDI [113]. Chen et al. engineered *S. boulardii* to secrete a single tetraspecific antibody that can effectively and extensively neutralize the main virulence factors of CDI (TcdA toxin and TcdB toxin), fighting the disease without causing antibiotic resistance. In the prevention and treatment experiments on mouse disease models, the engineered bacteria proved to have a protective effect on primary and recurrent CDI. This engineered yeast immunotherapy has the advantage of being able to be used in combination with antibiotics and has the potential to be used as a CDI risk prevention drug and a treatment drug for CDI patients [114]. Antibiotic-induced intestinal dysbiosis disrupts intestinal bile salt metabolism, which helps in the development of CDI [115]. Beyond antibody-based strategies, another approach targets intestinal microbiome and bile salt metabolism to combat CDI. Elvin Koh et al. postulated that targeting bile salt–metabolizing microbiome could disrupt CDI pathogenesis. They engineered EcN to restore gut bile salt metabolism during antibiotic-induced dysbiosis [116]. Engineered probiotics can detect sialic acid—a surrogate marker for dysbiosis—whose levels rise during antibiotic-induced gut dysbiosis [117]. They demonstrate that the engineered probiotics inhibited endospore germination and vegetative cell growth of *C. difficile in vitro*, and furthermore significantly mitigated CDI in mouse models [116]. Moreover, a team engineered *L. lactis* to secrete and extracellularly assemble a heterodimeric β-lactamase. β-lactamase-producing probiotics degrade the widely used broad-spectrum antibiotics β-lactams (penicillins, carbapenems, and cephalosporins) in the gut, thereby reducing antibiotic-induced intestinal dysbiosis and preventing the loss of colonization resistance against *C. difficile* [118].

***S. aureus*** is the main pathogen causing cardiovascular infection and bacteremia. *Staphylococcus aureus*, a multiantibiotic-resistant Gram-positive bacterium, is one of the leading causes of clinical and nosocomial infections [119], causing sepsis and severe skin infections, and is additionally the primary pathogen responsible for cardiovascular infections and bacteremia [120]. The detrimental effect of multidrug-resistant *S. aureus*, especially methicillin-resistant *S. aureus*, has even exceeded that of the human immunodeficiency virus [121]. In 2018, David Lubkowicz et al. designed *Lactobacillus* that can sense *S. aureus* using the quorum sensing system of *S. aureus*. The engineered probiotics can sense *S. aureus* through detect autoinducer peptide-I (AIP-I), a quorum sensing molecule produced by *Staphylococcus* sp. during pathogenesis [122]. After that, in 2023, Haoran Li et al. applying a similar *S. aureus* sensor system to *L. plantarum* and enabling it to release the killing protein lysostaphin into the medium after detection of AIP-I, effectively killing *S. aureus in vitro* [123].

***Listeria monocytogene*** (Lm) is a food-borne pathogen that primarily afflicts immune compromised individuals and can provoke septicaemia, meningitis and fetal infection or abortion in infected pregnant women [124]. A group of researchers engineered probiotic strains to express pathogen-specific surface proteins, preferentially bind to the host, thus preventing the pathogen from infecting the host [125]. Rishi Drolia et al. engineered *Lactobacillus* to express the Listeria adhesion protein (LAP) from a non-pathogenic *Listeria* (*L. innocua*) and a pathogenic *Listeria* (Lm) on the surface. This designer probiotics colonize the intestine, reduce Lm mucosal colonization and protect mice from lethal infection. At the same time, the LAP will stimulate the immune system of the host and enable the host to gain immunity to Lm. Bhunia's team engineered a LAP-producing *Lactobacillus casei* strain that can colonize the mouse intestine, competitively inhibit *Listeria* mucosal colonization and systemic spread, and protect the mice from lethal infection [126].

***Streptococcus pyogenes*** is a Gram-positive bacterium that is a common cause of throat infections and skin infections [127]. *Streptococcus salivarius K12* (SAL) is a probiotic strain of bacteria that has gained attention for its potential health benefits. It produces bacteriocins, which are antimicrobial substances that can inhibit the growth of other bacteria, including *Streptococcus* pyogenes [128]. Hackwon Do et al. found that the human oral probiotic SAL produces salivabactin, an antibiotic that effectively suppresses pathogenic GAS *in vitro* and in mice. However, actually SAL enhances GAS colonization in both mouse and human saliva. They inferred that GAS could eavesdrop on the peptide signals of quorum sensing between SAL cells and thus survive. They redesigned SAL to prevent the cross-activation of the GAS quorum sensing pathway and the degradation of SAL antimicrobials, and the engineered SAL could inhibit GAS colonization [129].

## 2 Challenges and future directions

Engineered probiotics can exert therapeutic effects through multiple mechanisms: (1) Secreting therapeutic or immunomodulatory factors like cytokines, enzymes, and antimicrobial peptides for targeted treatment. (2) Providing metabolic assistance to alleviate disorders such as PKU and hyperammonemia. (3) Colonizing and modulating the gut microenvironment by competing for nutrients, secreting SCFAs to regulate the immune system and maintain the intestinal barrier. (4) Enabling targeted oncology therapy by delivering anti-cancer agents and activating the immune system. Functionally, engineered probiotics act as delivery vehicles for therapeutic molecules, metabolic assistants, guardians of gut homeostasis, and precision-guided missiles targeting tumors, showcasing their versatility in addressing complex diseases.

In recent years, engineered probiotics have emerged as promising agents for modulating the gut-brain axis, a bidirectional pathway where the gastrointestinal tract and central nervous system interact. This axis integrates microbial, metabolic, and immunological signals to influence neurological processes, including mood regulation, cognitive function, and neurodegenerative disease progression [130]. Engineered probiotics can intervene in this axis through multiple strategies: for instance, by secreting neuroactive compounds such as gamma-aminobutyric acid (GABA) [131] or brain-derived neurotrophic factor (BDNF) [132] to directly affect neuronal activity.

With the help of synthetic biology tools, probiotic bacteria can be engineered to secrete therapeutic molecules or enzymes which made them perform specific therapeutic functions beyond natural counterparts [133]. For instance, CRISPR-Cas system is naturally occurring in *Bifidobacteria* and *Lactobacillus* [134], as these bacteria frequently encounter bacteriophage DNA or foreign plasmids in natural habitats like fermented foods and the gastrointestinal tract (GIT). When probiotic strains lack functional CRISPR-Cas systems, exogenous ones can be introduced via plasmid-based vectors. Recently, *B. licheniformis* with heterologously expressed nCas9 was used to knock out six chromosomal genes for functional study [135]. Zhou et al. conducted a study on seamless genome engineering using the heterologous type II CRISPR-Cas9 system and the endogenous type I-B CRISPR-Cas system in the probiotic *C. butyricum* [136]. Oh et al. successfully performed gene editing in *L*. *reuteri* by combining CRISPR-Cas9 with single-stranded DNA template recombination [137]. Therefore, the abundant genetic engineering tools will create different user-defined probiotic functions against various diseases. Future efforts will focus on developing genetic toolboxes for more effective delivery of therapeutic molecules and enzymes via human commensals such as *A. muciniphila*.

Besides the promising progresses in the field of engineered probiotics, there are still many concerns associated with the use of engineered probiotics as a therapeutic means: (1) Safety issues to human and environment: Long-term efficacy of programmed probiotics is unknown and the effect of their releases to environment is not systematically studied. Additionally, many of these engineered strains have been not clinically investigated in human subjects. (2) The mechanism of how probiotics achieve a protective effect is not fully answered. Although the effectiveness of recombinant probiotics has been demonstrated in various *vivo* models, some human experiments have not obtained similar results. The possible reason is that the human intestinal microbiome is more complex than animal models, and different dietary habits and intestinal microbiota structure may affect the activity of recombinant probiotics and thus affect their functions. The above-mentioned concerns therefore require more research efforts, to fully explore the field of engineered probiotics for disease treatments.

## Ethics approval and consent to participate

Not applicable.

## Consent for publication

Not applicable.

## Availability of data and materials

Not applicable.

## Authors' contributions

L.Z. and N.C. drafted the manuscript. H.C., C.T., J.W., Y.W., Y.Z. and H.G. assisted the manuscript write-up. J.Y. revised the manuscript.

## Funding

This work was supported by the National Key R&D Program of China (grant no. 2024YFC3407000), the 10.13039/501100001809National Natural Science Foundation of China (grant no. 32270087), the 10.13039/501100012226Fundamental Research Funds for the Central Universities (grant no. 20720240120), and ZhenSheng Biotech.

## Declaration of competing interest

The authors declare that they have no known competing financial interests or personal relationships that could have appeared to influence the work reported in this paper.

The author is an Editorial Board Member/Editor-in-Chief/Associate Editor/Guest Editor for this journal and was not involved in the editorial review or the decision to publish this article.
